# An optimization model of tram timetables considering various signal priority strategies

**DOI:** 10.1038/s41598-022-19762-9

**Published:** 2022-10-04

**Authors:** Jing He, Yuting Xu, Yanhuan Li, Jian Yang, Sihui Long

**Affiliations:** 1 Faculty of Transportation Engineering, Kunming University of Science and Technology, Kunming, 650500 China; 2 Lijiang Xueshan Rail Transit Co., Ltd., Lijiang, 674100 China

**Keywords:** Engineering, Mathematics and computing

## Abstract

Modern trams generally operate in a semi-independent Right Of Way that intersects with social vehicles at junctions. Typically, there are two signal priority strategies at junctions: active signal priority strategy and no-signal priority strategy. The active signal priority strategy is applied to improve the efficiency of the tram. However, it inevitably causes delays to social vehicles. The no-signal priority strategy could reduce the influence on social vehicles, but it will increase the tram travel time. Therefore, we develop a Mixed-Integer Linear Programming model to optimize the tram timetable and consider various signal priority strategies. In the model, the signal priority strategies of the tram are a set of decision variables that consider the traffic flow of social vehicles rather than fixed input parameters. The model considers minimizing the overall travel time of the tram and the negative utility of signal priority strategies. A numerical experiment is conducted to demonstrate the validity of the proposed model. The experimental results show that the proposed method can optimize the tram timetable and maximize the overall benefits of the junction. Moreover, we compare the experimental results of the proposed method with the approach of fixing the signal priority strategy for the tram at junctions. On the one hand, our proposed method can improve the operational efficiency of trams, i.e., the travel time decreases by 16.60%. On the other hand, the negative utility of signal priority for the comprehensive scheme proposed in this work reduces by 39.45%.

## Introduction

The modern tram system is an urban rail transit system with small and medium volumes. Trams have the outstanding advantages of being energy-efficient, environmentally friendly, fast, flexible in terms of transportation capacity, and comfortable. The development and implementation of trams in China have increased in recent years. By the end of 2021, trams in 20 cities (demonstration zones) on the Chinese mainland had already been put into operation, with a total mileage of over 503.60 km. Trams in China are entering a period of rapid development.

Modern trams generally operate in a semi-independent right of way (ROW), which intersects with social vehicles at junctions. The signal control and speed limits at junctions have a significant impact on the operation process of the tram. The trams may stop at a red light at the junction, which results in an extra start-stop time. To enhance the operational efficiency of trams, the current method is to set up a Transit Signal Priority (TSP) at signalized junctions to provide an extra green duration for trams. There are two forms of TSP: a passive signal priority strategy and an active signal priority strategy. Passive signal priority adjusts the signal timing parameters at the signalized junctions along the line so that trams running at the designed speed can pass through all junctions without stopping. However, owing to the interruption of the station and junction, the longer boarding times than scheduled may lead the pre-set green wave band to fail. If the tram still runs at the designed speed, it may stop at the red light at the junction.

The active signal priority differs from the passive signal priority. It can obtain information about trams through detectors. Then we determine whether to give the trams priority pass time according to the detection result. When a tram approaches the junction, the signal timing is adjusted according to the pre-set priority logic to prioritize the trams to pass if the signal priority strategy is activated. In addition to TSP, tram timetable optimization is another method for reducing the number of tram stops at junctions. This method adjusts the section running time and dwell time of trams so that trams reach junctions in the green phase. However, the adjustment range of the timetables is limited. The speed limit and the number of passengers boarding and alighting constrain the minimum section running time and dwell time of trams. The maximum section running and dwell times were also defined to maintain an equitable service level. Consequently, optimizing the timetables cannot guarantee that all trams arrive at junctions during the green phase.

Compared to the method that merely optimizes the tram timetable or solitary TSP, coupled optimization of the tram timetable and TSP may further reduce the tram travel time and improve the operational efficiency of the tram. However, in practice, trams intersect with social vehicles at junctions. Although the TSP method has the potential to increase the operational efficiency of a tram, the excessive signal adjustment will cause massive delays and negative impacts on social vehicles. This effect, particularly during peak commuting hours, can cause severe road traffic congestion. Therefore, reasonably combining the TSP control approach to construct an efficient timetable is significant for maximizing the overall benefits of junctions.

Therefore, this research aims to create a comprehensive model to minimize the overall travel time of trams and the negative utility of signal priority for social vehicles. We develop a Mixed-Integer Linear Programming (MILP) model to optimize the tram timetable and consider various signal priority strategies. In the model, the signal priority strategies of the tram are a set of decision variables that consider the traffic flow of social vehicles rather than the fixed input parameters. A numerical experiment is conducted to demonstrate the validity of the proposed model.

The remainder of this study is organized as follows. Section "Literature review" elaborates on the literature review on the problem of tram timetable optimization and TSP actions. Section "Problem description" presents the problem of adopting different signal priority strategies during different travel periods. Section "Mathematical model" constructs a model to optimize the tram timetable to minimize the travel time of trams and the negative utility of signal priority for social vehicles. Section "Mathematical model" further develops methods for solving the proposed model. Section "Numerical experiment" presents numerical experiments to test the validity and feasibility of the developed model. Section "Conclusions and future research" presents the main results of this study and offers possibilities for future research.

## Literature review

### Research on train timetables

As an essential component of urban rail transit operation organizations, train timetables determine the arrival and departure times of the trains at each station and play a crucial role in managing and operating urban rail transit systems^1,2^. Most of the current research on train timetable optimization has focused on railway and metro transportation. Nachtigall et al.^3–7^ proposed a model for timetable optimization to reduce passenger waiting times at stations. Motvallian et al.^8^ developed a model that considers the tram operation time and the number of passengers who successfully reached their destination. A heuristic approach based on a genetic algorithm was used to address this problem. Furthermore, several academics have researched timetable optimization problems from the perspective of corporate interests. To increase the interest of operating companies, Caprara et al.^9,10^ proposed a model containing Lagrangian relaxation to maximize corporate operations. Higgins et al.^11^ built a train timetable optimization model to reduce overall train delays and operating costs. Zhu et al.^12^ considered the conflict in benefits between operating companies and passengers. They analyzed the relationship between the train departure headway and departure time of passenger choice. Subsequently, a two-level planning model was proposed and solved using a two-stage genetic algorithm.

Compared with the running process of other forms of rail transit systems, modern trams generally operate in semi-independent ROW that intersects with social vehicles at junctions. The method for optimizing the train timetable of the subway under exclusive rights-of-way cannot be directly applied to tram systems. Therefore, some scholars have researched the optimization of tram timetables. Ji et al.^13^ developed a mixed integer model to resynchronize traffic signal timings to guarantee tram movements. Jiang et al.^14^ proposed a model to optimize the tram timetable under passive adjustment of signal timing at junctions to minimize tram travel time. Zhou et al.^15^ considered the effect of an operation diagram on signal priority. They proposed a strict operation diagram constraint model, HT-TRAM, and a loose operation diagram constraint model, ST-TRAM. Ji et al.^16^ developed a method for optimizing a tram timetable. This method can reduce the tram section running time while increasing the robustness of timetables. In addition, some scholars have developed tram timetable research that considers tram manipulation behavior. Li et al.^17^ considered the influence of train manipulation on energy consumption. They developed a two-layer joint optimization model of tram timetables and energy-efficient operations to minimize traction energy consumption. A bi-level genetic to solve the model. Zhang et al.^18^ constructed an energy-efficient optimization model guided by the section speed of trams to reduce the tram travel time and total energy usage.

### Research on transit signal priority

Wang et al.^19^ divided the priority control of trams into two categories: time priority and space priority. Shalaby et al.^20^ took the tram operated by King Street in Toronto as an example and conducted micro-simulation experiments for four scenarios. It includes unconditional signal priority, no-signal priority, no left turn, and no other traffic operation. They summarized the advantages of the above four scenarios. Zhong et al.^21^ studied the signal coordination of the tram line and realized the relative priority control of the signal at the junction of the tram. TSP approaches primarily utilize either a passive priority strategy or an active priority strategy^22,23^. Sermpis et al.^24^ described the Athens tram signal priority scheme and compared the implementation effects of passive signal priority strategy and active signal priority strategy. Chen et al.^25^ obtained a passive green wave coordinated control strategy based offline by analyzing the running characteristics of the tram and simulating the signal system. The method improves the punctuality of the trams. Ou et al.^26^ considered the tram characteristics and signal priority control at junctions and constructed a tram speed guidance model. The model was aimed at reducing the tram delays at the junctions. Ji et al.^27^ proposed three control strategies—green extension, vehicle hold, and speed steering—to guide the tram in the proper course to improve its reliability of the tram. Zhou et al.^28^ used vehicle delay and junction saturation as constraints. Then, the priority time thresholds of the two signal priority strategies of green extension and early green are determined. This study was conducted using the Webster graphic method. Wang, Y.-P et al.^29,30^ considered the traffic demand of trams and social vehicles and proposed an active signal priority control model for trams based on deep reinforcement learning.

Some academics have studied signal priority strategy and tram timetable design. Shi et al.^31,32^ proposed a joint optimization model for tram timetables and transit signal priority. It aimed to decrease the overall travel time of trams and reduce the negative utility of signal priority on junctions. Zhang et al.^33^ proposed a single-line timetable optimization method for two-way trams based on an active signal priority strategy based on^31,32^. Zhou et al.^34^ established a collaborative optimization model for tram timetables and junction signal timing to maximize the overall benefits of trams and social vehicles. Jeong et al.^35^ considered the effect of the red-light phase difference at the same junction on the operation of trams. They proposed a tram passive priority control model based on the mixed-integer linear programming green waveband optimization model MAX BAND to ensure the green wave passage of arterial social vehicles simultaneously under the condition of fundamental tram passage green wave bandwidth. Zhang et al.^36^ proposed an asymmetric and unequal-width trunk signal coordination model AM-BAND based on the trunk signal coordination model MAX BAND^37–39^ and the unequal-width trunk signal coordination model MULTIBAND^40,41^. BAM-TRAMBAND is a model for artery signal coordination optimization proposed by Zhou et al.^42^. The Asymmetrical Multi-BAND (AM-BAND) approach was used to develop it. The tram line in Ningbo, China, validated the proposed model. Based on the literature^42^, Bai et al.^43,44^ fixed the green wave bandwidth of trams and built a passive signal priority model. It was aimed at maximizing the green-wave bandwidth of social vehicles to reduce vehicle delays.

Research on combining a signal priority strategy and tram timetables provides a good idea for tram timetables optimization. However, the research is primarily limited to a method that adopts a fixed signal priority strategy to optimize timetables. It neglects the impact on the optimization of tram timetables by adopting different signal priority strategies during different travel periods. Therefore, a reasonable combination of signal priority strategies to design an efficient timetable is a pressing issue that needs to be solved.

The contributions of this study are as follows:

- This study comprehensively optimizes the travel time of a tram and the negative utility of signal priority strategies from a systems perspective. Collaborative optimization can maximize the overall benefits of trams and social vehicles and provide theoretical reference and data support for tram operators in timetable design.
- This study proposes a method to optimize the tram timetable by adopting different signal priority strategies during different travel periods. Compared with the current method that utilizes a fixed signal priority strategy to optimize tram timetables, we concentrate on the impact on tram timetable optimization of adopting different signal priority strategies during different travel periods. In the model, the signal priority strategies of the tram are a set of decision variables considering the traffic flow of social vehicles rather than the fixed input parameters. A numerical experiment is conducted to show the validity of the proposed model.
- This study develops a Mixed-Integer Linear Programming (MILP) model. The model effectively minimized the overall travel time of the tram and the negative utility of signal priority for social vehicles. Moreover, the various commercial solvers can solve the bi-objective MILP optimization model in this study.

## Problem description

### Problem description

**Figure 1:**
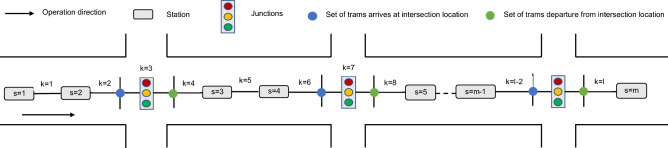
Schematic diagram of a tram line.

Modern trams generally operate in semi-exclusive ROW that intersects with social vehicles at junctions. It passes through several signalized junctions during the operation. According to the topological structure relationship between stations and junctions, the tram operating process is split into multiple operation sections, as indicated in Fig. 1. To facilitate the model characterization, we portray the junctions along the route as virtual stations. The set of stations and junctions is $S=\left\{\left.\mathrm{1,2},\cdots m-1,m\right\}\right.$, and the set of operation sections is $K=\left\{\left.\mathrm{1,2},\cdots ,l-1,l\right\}\right.$.

The primary purpose of tram timetable optimization is to reduce the overall travel time of trams, which is achieved by adjusting the section running time and dwell time of trams. Because periodic changes characterize signalized junctions, even if the trams run fast, there is no guarantee that they can pass through junctions without stopping. To ensure the smooth passage of the trams through the junctions and improve the operational efficiency, the existing method usually only adopts a fixed signal priority strategy to optimize timetables. However, they ignored the impact of adopting different signal priority strategies during the different travel periods on the tram timetables optimization results.

**Figure 2:**
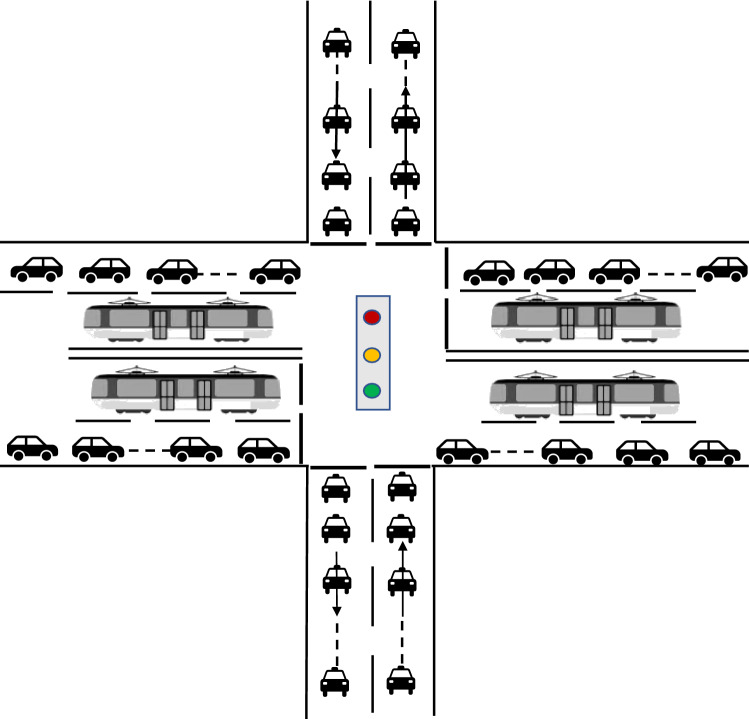
Peak hour traffic.

**Figure 3:**
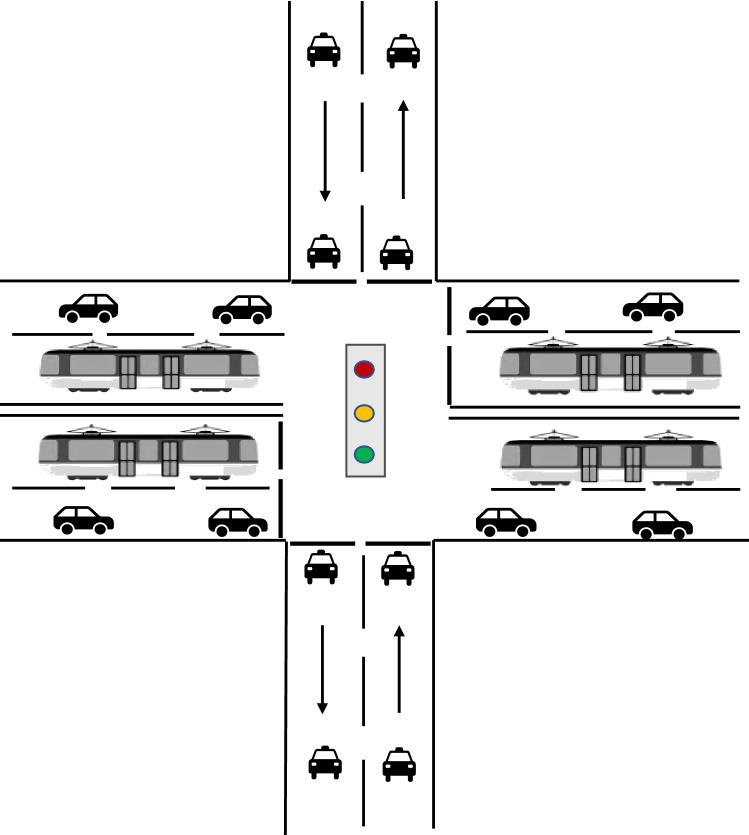
Off-peak hour traffic.

By detecting the actual traffic flow at the junction, we found that the traffic flow of social vehicles at each phase is higher during peak commuting hours, as shown in Fig. 2. If the no-signal priority strategy is adopted for the tram phases during this period, there is no need to sacrifice the green duration of the other phases, and the negative impact on social vehicles is smaller. During off-peak hours, the traffic flow of social vehicles in each phase was smaller, as shown in Fig. 3. If an active signal priority strategy is adopted for the tram phases during this period, trams can be guaranteed to pass through junctions at the green light without stopping. The total travel time of trams was reduced. The above analysis demonstrates that we can determine the signal priority strategy adopted by trams by detecting the traffic flow of social vehicles during different travel periods. This method can minimize the overall travel time of trams and the negative utility of signal priority for social vehicles.

In summary, this study takes the operation process of trams during different travel periods as the research object and constructs an approach to optimize tram timetables by adopting different signal priority strategies during different travel periods. The goal is to minimize the overall travel time of trams and the negative utility of signal priority for social vehicles.

### Assumptions

#### Assumption 1

The tram runs reliably. In this paper, trams possess exclusive rights-of-way (ROW) except for junctions and assured tram movements at junctions. Consequently, random events do not disrupt tram movements.

#### Assumption 2

The original signal timing at each junction is optimal, and the negative utility per second of the adjustment made when using the active signal priority strategy is known. This assumption can also be found in recent timetable research, such as Shi^16^.

#### Assumption 3

By fine-tuning the existing signal timing, extending the dwell time, controlling the tram section running time, or using an active signal priority strategy, the trams can cross the junction without stopping. Consequently, the trams had no delays at the junctions.

#### Assumption 4

The timetable optimization process does not change the departure time of the trams at the first station.

#### Assumption 5

The problem of several train lines sharing the same track is not considered.

#### Assumption 6

The signal cycle length is the same for each junction during different travel periods.

### Notations

Table 1 presents all of the relevant notations utilized in the formulation.

**Table 1:** Notations.

Notations	Description
**Sets**	
$Q$	Set of trams,$Q=\left\{\mathrm{1,2},\cdots r-1,\left.r\right\}\right.$
$S$	Set of stations and junctions,$S=\left\{\mathrm{1,2},\cdots m-1,\left.m\right\}\right.$
$\widetilde{S}$	Set of stations,$\widetilde{S}\subset S$
${S}^{^{\prime}}$	Set of entrances to junctions,${S}^{^{\prime}}\subset S$
${S}^{^{\prime\prime} }$	Set of exits at junctions,${S}^{^{\prime\prime} }\subset S$
$K$	Set of sections,$K=\left\{\mathrm{1,2},\cdots l-1,\left.l\right\}\right.$
$B$	Set of tram travel periods,$B=\left\{\left.\mathrm{1,2},\cdots y-1,y\right\}\right.$
**Indexes**	
$q$	Index of trams,$q\in Q$
$s$	Index of stations or junctions,$s\in S$
$k$	Index of sections,$k\in K$
$b$	Index of tram travel periods,$b\in B$
**Parameters**	
${c}_{s}$	Signal cycle length at junctions $s$,$s\in {S}^{^{\prime}}$
${o}_{s}$	Signal offset at junction $s$ (compared with the first junction in the “downward” direction),$s\in {S}^{^{\prime}}$
${T}_{s}^{g}$	Green duration of tram phase at junction $s$,$s\in {S}^{^{\prime}}$
${t}_{q,s}^{{W}_{0}}$	The initial dwell time of tram $q$
${T}_{s}^{r}$	Red duration of tram phase at approaching junctions $s$,$s\in {S}^{^{\prime}}$
${t}_{k}^{min}$	The lower limit of the running time in section $k$ under non-stop movements at the junction
${t}_{k}^{max}$	The upper limit of the running time in section $k$ under non-stop movements at the junction
$t_{k}^{{min^{\prime}}}$	The lower limit of the running time in section $k$ under junction stop movements
$t_{k}^{{max^{\prime}}}$	The upper limit of the running time in section $k$ under junction stop movements
${G}_{q}$	The departure time of tram $q$ at the first station
${H}_{A}^{A}$	The minimum headway between two consecutive train arrivals at the same station
${H}_{D}^{D}$	The minimum headway between two consecutive trains departures from the same station
${H}_{D}^{A}$	The minimum headway between a train departure and another train arrival at the same station
${W}_{s}$	Unit negative utility (per second) of adopting signal priority strategy at junction $s$,$s\in {S}^{^{\prime}}$
${\theta }_{b}^{s}$	The traffic flow of social vehicles
**Decision variables**	
${t}_{q,k}$	Time of tram $q$ in running section $k$
${t}_{q,s}^{W}$	Dwell time of tram $q$ in station $s$,$s\in S$
${t}_{q,s}^{D}$	The departure time of tram $q$ from station $s$,$s\in S$
${t}_{q,s}^{A}$	The arrival time of tram $q$ at station $s$,$s\in S$
${t}_{q,s}^{ot}$	Time spent in a signal cycle when tram $q$ arrives at junction $s$, $s\in {S}^{^{\prime}}$
${t}_{q,s}^{rt}$	Time remaining in a signal cycle when tram $q$ arrives at junction $s$,$s\in {S}^{^{\prime}}$
${\alpha }_{q,b,s}$	A binary variable, which is 1 if tram $q$ stops at junction $s$ during period $\mathrm{b}$; 0, otherwise
${u}_{q,s}$	Signal cycle number of tram $q$ to the stop line at junction $s$,$s\in {S}^{^{\prime}}$
${\beta }_{b}^{s}$	A binary variable; which is 1 if the no-signal priority strategy is adopted at the junction $s$; 0, otherwise
${I}_{q,b,s}$	The negative utility of adopting the signal priority strategy

## Mathematical model

### Constraints related to tram timetables

1. Section Operation

Compared to a tram that can pass through the junctions without stopping, it will have an extra start-stop time when the tram stops at a red light at the junction. Therefore, whether a tram stops at the junction affects the running time of the section adjacent to the junction. In peak hour b, if the tram arrives at junction stop line $s+1$ during the green phase, the lowest limit of the running time of the section is ${t}_{k}^{min}$. The running time of the section had an upper limit of ${t}_{k}^{max}$. If the tram arrives at junction stop line $s+1$ during the red phase, it must stop at the junction to wait for red signals. The lowest limit of running time of the section is $t^{{{_{k}{min^{\prime } }} }}$. The running time of the section had an upper limit of $t_{k}^{{max^{\prime } }}$. In off-peak hour b, the lowest limit of the running time of the section is ${t}_{k}^{min}$. The running time of the section had an upper limit of ${t}_{k}^{max}$.

1 $$ \begin{array}{*{20}l} {\left\{ {\begin{array}{*{20}c} {t_{q,k} \ge \left( {1 - \alpha_{q,b,s} - \alpha_{q,b,s + 1} } \right)t_{k}^{\min } + \left( {\alpha_{q,b,s} + \alpha_{q,b,s + 1} } \right)t_{k}^{{min^{\prime } }} } \\ {t_{q,k} \le \left( {1 - \alpha_{q,b,s} - \alpha_{q,b,s + 1} } \right)t_{k}^{\max } + \left( {\alpha_{q,b,s} + \alpha_{q,b,s + 1} } \right)t_{k}^{{max^{\prime } }} } \\ \end{array} } \right.} \hfill \\ {\forall q \in Q,k \in K,s \in S\backslash \left\{ {\left. m \right\}} \right.,\alpha_{q,b,s} \in \left\{ {0,\left. 1 \right\}} \right.} \hfill \\ \end{array} $$

(2) Dwell Time

2 $$ t_{q,s}^{{W_{0} }} \le t_{q,s}^{W} \le \left( {t_{q,s}^{{W_{0} }} + T_{s}^{r} } \right) \forall q \in Q,s \in \tilde{S} $$

$$ \begin{gathered} \beta_{b}^{s} = 1,\left\{ {\begin{array}{*{20}c} {t_{q,s}^{rt} + \left( {\alpha_{q,b,s} - 1} \right)M \le t_{q,s}^{W} \le t_{q,s}^{rt} + \left( {1 - \alpha_{q,b,s} } \right)M} \\ { - \alpha_{q,b,s} M \le t_{q,s}^{W} \le \alpha_{q,b,s} M} \\ \end{array} } \right. \hfill \\ \beta_{b}^{s} = 0,t_{q,s}^{W} = 0 \hfill \\ \end{gathered} $$

3 $$\forall q\in Q,s\in {S}^{^{\prime}},b\in B,{\alpha }_{q,b,s}\in \left\{0,\left.1\right\}\right.,{\beta }_{b}^{s}\in \left\{0,\left.1\right\}\right.$$

4 $${t}_{q,s}^{W}=0 \, \forall q\in Q,s\in {S}^{^{\prime\prime} },b\in B$$

Constraint (2) indicates that the minimum dwell time is the initially scheduled dwell time. The maximum dwell time is calculated by adding the minimum dwell times and the red duration adjacent to the junction. Constraint (3) indicates that if the tram stops at the junction, the tram dwell time at the junction is the time when the red light changes to green. The tram dwell time at the junctions is zero if it can pass through the junctions under green light without stopping. Constraint (4) indicates that the dwell time “departure from the junction location” is zero.

(3) Tram Arrival and Departure Time

The departure time is calculated by summing the running time of the last station and the dwell time of the station. The arrival time is calculated by summing the running time and dwell time of the previous station.

5 $${t}_{q,1}^{D}={G}_{q}+{t}_{q,1}^{W} \, \forall q\in Q$$

6 $$ t_{q,s}^{D} = G_{q} + \mathop \sum \limits_{k = 1}^{s - 1} t_{q,k} + \mathop \sum \limits_{s = 1}^{s} t_{q,s}^{W} { }\forall q \in Q,s \in S\backslash \left\{ {\left. 1 \right\}} \right. $$

7 $$ \begin{aligned} t_{q,s}^{A} = & G_{q} + \mathop \sum \limits_{k = 1}^{s - 1} t_{q,k} + \mathop \sum \limits_{s = 1}^{s - 1} t_{q,s}^{W} { } \\ { } & \forall q \in Q,s \in S\backslash \left\{ {\left. 1 \right\}} \right. \\ \end{aligned} $$

(4) Selection of signal priority strategy

This method determines the signal priority strategy adopted by the tram by detecting the traffic flow of social vehicles during different travel periods. According to relevant literature^45–47^ and the actual measurement of traffic flow at junctions, we select the number of social vehicles as 800 as the threshold for determining which signal priority strategy to adopt. If the traffic flow of social vehicles is ≥ 800 during the travel period, the no-signal priority strategy is adopted; otherwise, the active signal priority strategy is adopted.

8 $$ \begin{aligned} \beta_{b}^{s} = & \left\{ {\begin{array}{*{20}c} {1,if \theta_{b}^{s} \ge 800} \\ {0,otherwise} \\ \end{array} } \right. \\ & \forall s \in S^{\prime},b \in B \\ \end{aligned} $$

(5) Determination of whether a tram needs to stop at the junctions.

When the tram arrives at the signalized junctions, if the time consumed in the signal cycle is less than the green duration of the tram phase, it can pass through the junctions without stopping. Otherwise, it must be stopped at the junction.

9 $$ \left\{ {\begin{array}{*{20}l} {C_{s} \left( {u_{q,s} - 1} \right) < t_{q,s}^{A} - o_{s} } \hfill \\ {C_{s} u_{q,s} \ge t_{q,s}^{A} - o_{s} } \hfill \\ \end{array} } \right. \forall q \in Q,s \in S^{\prime } $$

10 $$ t_{q,s}^{ot} = t_{q,s}^{A} - o_{s} - C_{s} \left( {u_{q,s} - 1} \right){ }\forall q \in Q,s \in S^{\prime } $$

11 $$ t_{q,s}^{rt} = C_{s} - t_{q,s}^{ot} \forall q \in Q,s \in S^{\prime } $$

12 $$ \begin{aligned} \beta_{b}^{s} = & 1,\left( {\alpha_{q,b,s} - 1} \right)M < t_{q,s}^{ot} - T_{s}^{g} \le \alpha_{q,b,s} M \\ \beta_{b}^{s} = & 0,\alpha_{q,b,s} = 0 \\ & \forall q \in Q,s \in S^{\prime } ,b \in B \\ \end{aligned} $$

**Figure 4:**
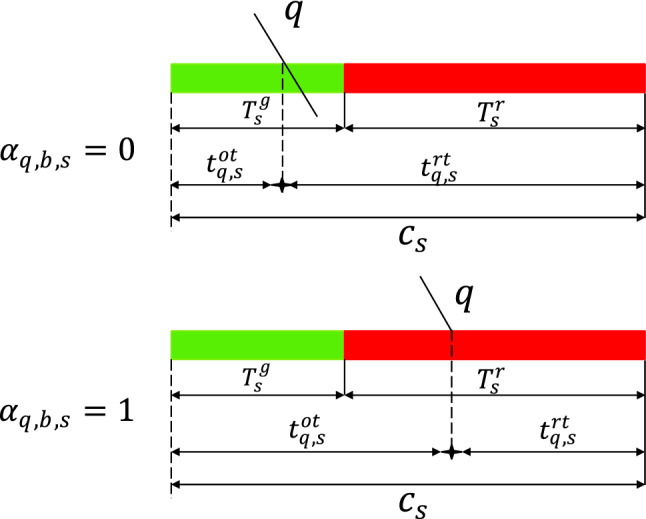
Consumption time and remaining time.

**Figure 5:**
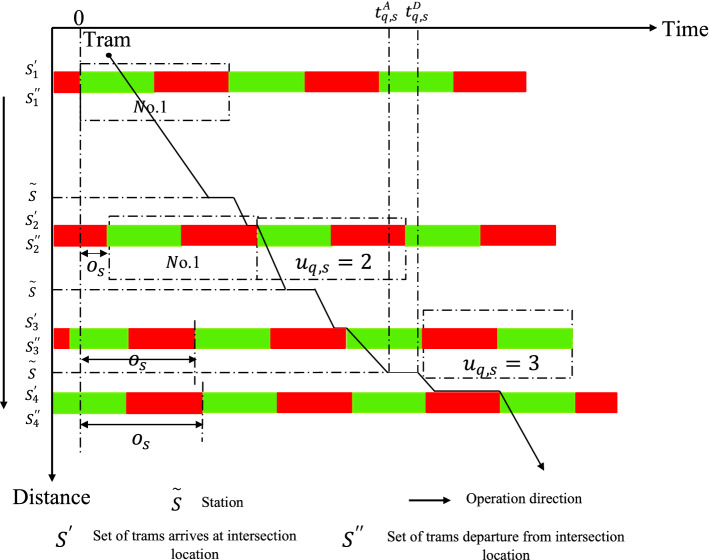
Offsets and signal cycle numbers.

As shown in Fig. 4.、Fig. 5, constraint (9) is the number of signal cycles when the tram reaches the junction. Constraints (10) and (11) are the equations for the time spent and remaining in a signal cycle whenever tram $q$ reaches junction $s$. Constraint (12) is the determination constraint for whether tram $q$ must stop at junction $s$.

(6) The negative utility of signal priority strategy

When the tram passes through the junctions during the green phase, it inevitably causes delays in social vehicles. The negative utility of adopting the signal priority strategy is quantified as the product of the signal priority response time, the negative utility of signal priority per unit time, and the traffic flow of social vehicles.

13 $$ \begin{aligned} I_{q,b,s} = & T_{s}^{g} \times W_{s} \times \theta_{b}^{s} \times \left( {1 - \alpha_{q,b,s} } \right) \\ & \forall q \in Q,{ }s \in S^{\prime},b \in B,\alpha_{q,b,s} \in \left\{ {0,} \right.\left. 1 \right\} \\ \end{aligned} $$

(7) Headways

14 $$ \begin{gathered} \left\{ {\begin{array}{*{20}c} {t_{q + 1,s}^{A} - t_{q,s}^{A} \ge H_{A}^{A} } \\ {t_{q + 1,s}^{A} - t_{q,s}^{D} \ge H_{D}^{A} } \\ {t_{q + 1,s}^{D} - t_{q,s}^{D} \ge H_{D}^{D} } \\ \end{array} } \right. \hfill \\ \forall q \in Q\backslash \left\{ {\left. {\left| r \right|} \right\}} \right.,s \in S \hfill \\ \end{gathered} $$

### Objective function

This paper proposes a method to optimize tram timetables by adopting different signal priority strategies during different travel periods to address the mentioned issues. The objective function consists of two parts. Part 1 is the total travel time of all trams. The second part is the total negative utility of the signal priority strategy. The specific form of the objective function is as follows:

15 $$\mathit{min}{\sum }_{q\in Q}{\sum }_{k\in K}{\sum }_{s\in S}\left({t}_{q,k}+{t}_{q,s}^{W}\right)+{\sum }_{q\in Q}{\sum }_{s\in {S}^{^{\prime}}}{\sum }_{b\in B}{I}_{q,b,s}$$

subject to constraints (1)–(14).

### Solution approach

The optimization model constructed in this paper is a MILP model. It can be solved using the optimization solver GUROBI. A common method for solving multi-objective optimization problems is the linear weighted method. Its fundamental concepts are as follows:

- Construct a single-objective optimization model
  Ignore the goal of minimizing the negative utility of the signal priority strategy and set a single-objective optimization model (marked as model $S1$) to minimize the travel time. The goal of this model is $min{z}_{1}$. Ignore the goal of minimizing the total travel time and set it to minimize the single-objective optimization model (marked as model $S2$) with the negative effect of the signal priority strategy as the goal. The goal of this model is $min{z}_{2}$.
- Solve single-objective optimization models
  Two single objective optimization models $S1$ and $S2$ are solved respectively, and the corresponding objective values of the optimal solutions are denoted as ${z}_{1}^{^{\prime}}$ and ${z}_{2}^{^{\prime}}$ respectively.
- Solve for the boundary points of the Pareto solution set
  Add the constraint $${z}_{1}<{z}_{1}^{^{\prime}}$$ to the constraints of the model $$S1$$. The obtained target value is recorded as $${z}_{1}^{*}$$, and the output value of the energy consumption index is recorded as $${z}_{2}^{*}$$. The solution $${z}_{1}^{*},{z}_{2}^{*}$$ is a boundary point in the Pareto solution set $$\Theta $$ of the dual-objective optimization problem. Similarly, add $${z}_{2}<{z}_{2}^{^{\prime}}$$ to the constraints of model $$S2$$, and solve to obtain another boundary point of the Pareto solution set, denoted as ($${z}_{1}^{**}$$、$${z}_{2}^{**}$$). Then set the solution ($${z}_{1}^{*}$$, $${z}_{2}^{*}$$) and the solution ( $${z}_{1}^{**}$$, $${z}_{2}^{**}$$) are added to the Pareto solution set $$\Theta $$.
- Set weights for two objectives
  The weights ${\alpha }_{1}$ and ${\alpha }_{2}$ of the total travel time and total negative utility are given respectively, satisfying ${\alpha }_{1}\ge 0$, ${\alpha }_{2}\ge 0$, ${\alpha }_{1}+{\alpha }_{2}=1$*.*
- Normalize the objective function
  Set $${\varphi }_{1}={\alpha }_{1}/{z}_{1}^{*},{\varphi }_{2}={\alpha }_{2}/{z}_{2}^{**}$$
- Construct a single-objective optimization model based on the linear weighting method
  The goal of the model is set as $minz=\mathrm{min}({\varphi }_{1}\cdot {Z}_{1}+{\varphi }_{2}\cdot {Z}_{2})$, and the constraint is the formula of Eqs. (1)–(14).
- Solve the single-objective optimization model based on the linear weighting method
  Solve the model and add the resulting Pareto solution to the Pareto solution set $\Theta$.
- Obtain the Pareto Frontier
  Repeat steps 2–5 until the required number of Pareto optimal solutions are obtained and output the Pareto frontier according to the objective function value of the Pareto solution.

## Numerical experiment

We designed numerical experiments with a tram line as the background to evaluate the validity and feasibility of the model developed in this paper. We selected a weekday period of 7: 00–12: 00 to optimize the tram timetables using the proposed method.

### Numerical conditions and model parameters

#### Numerical conditions

**Figure 6:**
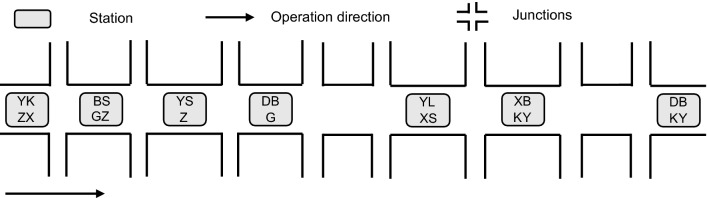
Schematic diagram of the line.

We designed the numerical experiments with a tram line as the background for this paper. The specific data are as follows. The tram line has seven stations and eight signalized junctions, and the tram line is shown in Fig. 6. To evaluate the validity and feasibility of the model developed in this work, we designed two other schemes for comparison.

All numerical experiments were performed on a computer with Intel (R) Core (TM) i7-1065G7 CPU, 8G memory, operating system win10, and solved using GUROBI.

### Model parameters

The range for section running time is shown in Table 2.

**Table 2:** The range for section running time.

No	Section	The range for running time under non-stop movements at the junction(s) (min, max)	The range for running time under stop movements at the junction(s) (min, max)
1	YKZX- YB1	(44, 77)	(47, 80)
2	YB1- YB2	(6, 6)	(8, 8)
3	YB2- BSGZ	(20, 20)	(24, 24)
4	BSGZ- BW1	(35, 62)	(40, 65)
5	BW1- BW2	(6, 6)	(8, 8)
6	BW2- YSZ	(20, 20)	(22, 22)
7	YSZ- XY1	(36, 66)	(41, 69)
8	XY1- XY2	(7, 7)	(9, 9)
9	XY2- DBG	(22, 22)	(24, 24)
10	DBG- YM1	(38, 65)	(42, 68)
11	YM1- YM2	(6, 6)	(9, 9)
12	YM2- YG1	(28, 52)	(33, 55)
13	YG1- YG2	(6, 6)	(9, 9)
14	YG2- YLXS	(20, 20)	(24, 24)
15	YLXS- YY1	(36, 66)	(41, 69)
16	YY1- YY2	(7, 7)	(9, 9)
17	YY2- XBKY	(20, 20)	(22, 22)
18	XBKY- ZH1	(31, 53)	(36, 56)
19	ZH1- ZH2	(6, 6)	(8, 8)
20	ZH2- SF1	(29, 56)	(34, 59)
21	SF1- SF2	(7, 7)	(9, 9)
22	SF2- DBKY	(22, 22)	(24, 24)

Tables 3 and 4 present the range for dwell time and the departure time at the first station.

**Table 3:** The range for dwell time.

No	Station	Range of dwell time(s) (min, max)
1	YKZX	(0, 80)
2	BSGZ	(25, 99)
3	YSZ	(38, 120)
4	DBG	(26, 101)
5	YLXS	(26, 101)
6	XBKY	(25, 91)
7	DBKY	(42, 50)

**Table 4:** The departure time at the first station.

No	Departure time	No	Departure time
1	7:04	11	9:29
2	7:19	12	9:44
3	7:33	13	9:58
4	7:48	14	10:13
5	8:02	15	10:27
6	8:17	16	10:42
7	8:31	17	10:56
8	8:46	18	11:11
9	9:00	19	11:25
10	9:15	20	11:40

The signal timing parameters for each signal junction during the study period are listed in Table 5. Table 6 describes the traffic flow of social vehicles at each junction during the different travel periods. The calculation of the negative utility of signal priority is inextricably linked to the traffic flow and junction saturation. The calculation process is extremely complicated. To simplify the problem, we refer to the literature^16,17^. The unit negative utility (per second) of adopting a signal priority strategy at a junction is ${W}_{s}=10$. The minimum headway between two consecutive train arrivals at the same station is ${H}_{A}^{A}=30$. The minimum headway between two consecutive train departures from the same station is ${H}_{D}^{D}=20$. The minimum headway between train departure and another train arrival at the same station is ${H}_{D}^{A}=30$.

**Table 5:** Signal timing data at signalized junctions.

No	Signal cycle(s)	Green time(s)	Red time(s)	Offset(s)
1	109	33	76	0
2	109	44	65	50
3	109	35	74	13
4	109	34	75	68
5	109	50	59	85
6	109	34	75	68
7	105	39	66	68
8	105	20	85	89

**Table 6:** The traffic flow of the social vehicles.

Junctions travel periods	2	5	8	11	13	16	19	21
7:00–7:30	500	461	428	488	483	452	487	498
7:30–8:00	511	531	500	501	571	529	514	544
8:00–8:30	1014	1419	931	974	1575	1271	964	1682
8:30–9:00	1081	1462	940	949	1605	952	1359	1552
9:00–9:30	607	651	611	604	612	631	652	617
9:30–10:00	656	633	629	600	500	495	571	780
10:00–10:30	517	513	537	548	520	541	532	580
10:30–11:00	924	1531	905	921	1582	1388	1548	1625
11:00–11:30	957	1629	950	1032	1592	1432	961	1584
11:30–12:00	921	1598	1269	1499	1636	920	1642	1569

### Results and discussions

#### Complexity analysis

The model constructed in this paper comprised continuous and binary variables. The magnitude of a set determines the size of each variable. Table 7 presents a complex analysis of the problem.

**Table 7:** Problem complexity.

Type	Variables of constraints	Theoretical dimension	Counts
Continuous variables	${t}_{q, k}$	$\sum \left|K\right|\cdot \left|Q\right|$	440
	${t}_{q, s}^{W}$, ${t}_{q,s}^{D}$,${t}_{q,s}^{A}$	$\sum \left|S\right|\cdot \left|Q\right|$	460
	${t}_{q,s}^{ot},$ ${t}_{q,s}^{rt}$,${u}_{q,s},$	$\sum \left| {S^{\prime}} \right| \cdot \left| Q \right|$	160
Binary	${\alpha }_{q,b,s}$	$\sum \left|S\right|\cdot \left|Q\right|$	460
	${\theta }_{b}^{s},$ ${\beta }_{b}^{s}$	$\sum \left| {S^{\prime}} \right| \cdot \left| Q \right|$	160
Constraints	Constraints (1)	$\sum \left|K\right|\cdot \left|Q\right|$	440
	Constraints (2)	$\sum \left|\widetilde{S}\right|\cdot \left|Q\right|$	140
	Constraints (3)	$\sum \left| {S^{\prime}} \right| \cdot \left| Q \right|$	160
	Constraints (4)	$\sum \left| {S^{\prime\prime}} \right| \cdot \left| Q \right|$	160
	Constraints (5)	$\sum \left|Q\right|$	20
	Constraints (6)–(7)	$\sum \left|S\right|\cdot \left|Q\right|$	460
	Constraints (8)–(13)	$\sum \left| {S^{\prime}} \right| \cdot \left| Q \right|$	160
	Constraints (14)	$\sum \left|S\right|\cdot \left|Q\backslash \left\{\left|r\right|\right\}\right|$	437

As indicated in Table 7, the timetable optimization problem proposed in this paper is a mega-scale linear programming problem. It contains 2530 integer variables, 2900 binary variables, and approximately 10,000 constraints. The comprehensive optimization issue can be addressed using the optimization solver GUROBI.

### Comparative discussions of optimization results

**Figure 7:**
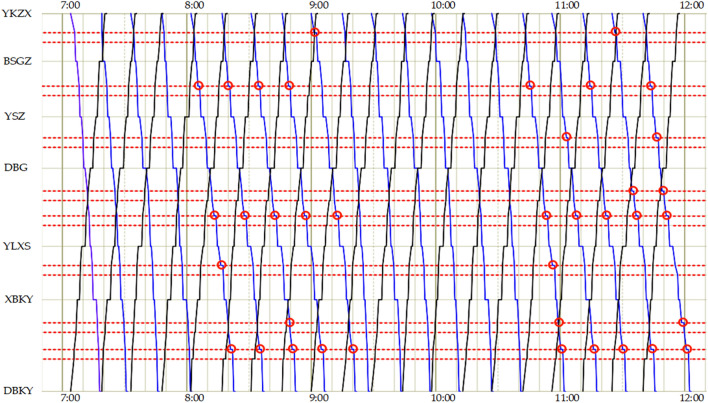
The tram timetable of comprehensive optimization scheme (this work).

According to the GUROBI data, the minimum overall travel time of trams after optimization is 21330 s, and the minimum negative utility of the signal priority strategy is 32,016,450. The operational efficiency of the line has enhanced significantly. Fig. 7 depicts the tram timetable for the comprehensive optimization scheme.

In Fig. 7, the red dotted lines represent the location of the junction, and the red circles indicate that the tram stops at the junction.

**Figure 8:**
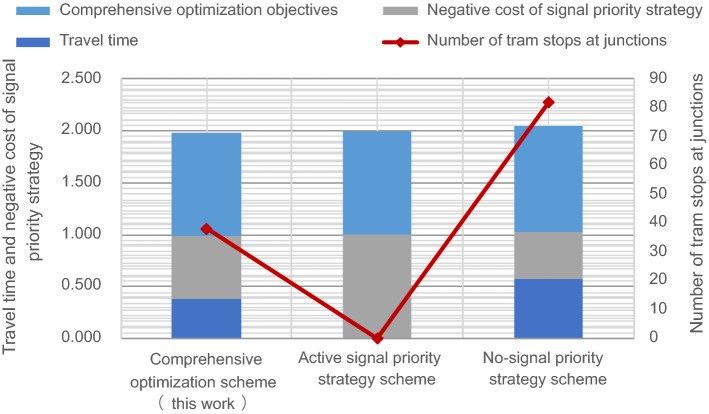
Comparison of comprehensive optimization objectives of trams for different schemes.

To evaluate the validity and feasibility of the model developed in this work, we designed two other schemes for comparison. Scheme 1 shows the adoption of the no-signal priority strategy for all travel periods. Scheme 2 shows the scheme for adopting the active signal priority strategy during all travel periods. Scheme 3 is the scheme proposed in this study that adopts different signal priority strategies under different travel periods. After normalizing the multi-objective function, a comparison of the optimization results for each scheme is shown in Fig. 8.

**Figure 9:**
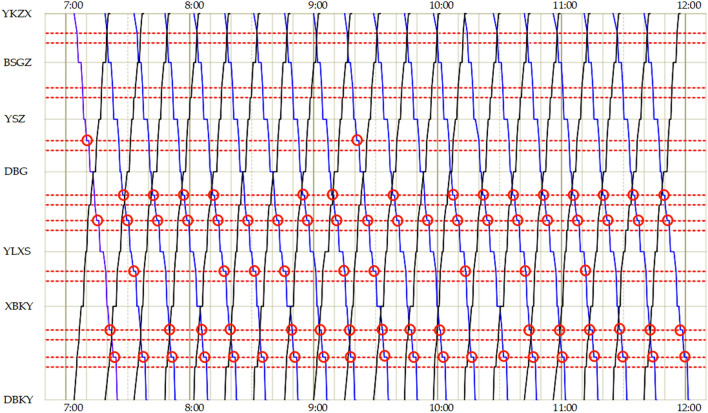
The tram timetable of the scheme for adopting the no-signal priority strategy scheme.

As shown in Fig. 8, compared with the scheme that only adopts the no-signal priority strategy, the travel time for the comprehensive scheme proposed in this study is reduced by 16.60%. The number of tram stops at junctions decreased by 53.66%. The above analysis revealed that when only adopting the no-signal priority strategy during all travel periods, trams do not always have priority to pass through junctions without stopping. These may be interrupted by a red signal at the junction. Consequently, the travel time for trams increased. The scheme proposed in this work can significantly decrease the number of tram stops at junctions, shorten overall tram travel time, and improve operational efficiency. The tram timetable for adopting the no-signal priority strategy scheme is shown in Fig. 9.

**Figure 10:**
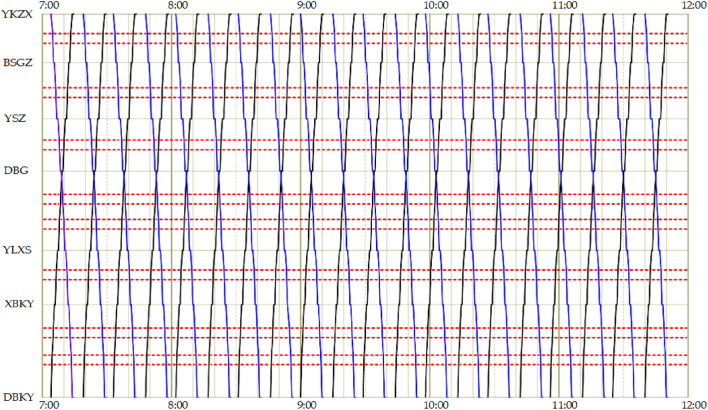
The tram timetable for adopting the active signal priority strategy scheme.

Compared with the scheme that only adopts the active signal priority strategy during all travel periods, the negative utility of signal priority for the comprehensive scheme proposed in this work decreased by 39.45%. The above analysis shows that although only adopting the active signal priority strategy can slightly shorten the overall travel time of the tram, the active signal priority strategy does not consider social vehicles. This inevitably causes delays in social vehicles. The scheme in this study decreases the negative utility of signal priority for the social vehicles by only slightly increasing tram travel times. It better balances the traffic benefits of tram and social vehicles to maximize the overall benefits of the junction. The tram timetable for adopting the active signal priority strategy scheme during all travel periods is shown in Fig. 10.

Based on the comparison results between the scheme in this study and the other two schemes, the subgoals of the scheme proposed in this work are slightly inferior to the optimal target values of each subgoal. However, overall, the comprehensive objective of the method developed in this study is optimal among the three schemes. The comprehensive method proposed in this work can optimize the tram timetable and balance the operational efficiency of the tram and social vehicles. Moreover, it can realize the purpose of providing theoretical references and data support for tram operators in designing timetables.

## Conclusions and future research

Modern trams generally operate in a semi-independent ROW that intersects with social vehicles at junctions. The trams may stop at a red light at the junction, which results in an extra start-stop time. To decrease the tram travel time and the number of tram stops, the Transit Signal Priority (TSP) is applied to provide an extra green duration for trams at the junctions. Although the active signal priority strategy has the potential to increase the operational efficiency of the tram, the excessive signal adjustment can cause massive delays and negative impacts on social vehicles. This effect, particularly during peak commuting hours, can cause severe road traffic congestion. Therefore, we develop a MILP model to optimize the tram timetable and consider various signal priority strategies. In the model, the signal priority strategies of the tram are a set of decision variables that consider the traffic flow of social vehicles rather than the fixed input parameters. This method can address the issues of low operating efficiency and a lack of consideration of the negative impact of the TSP on the junctions.

The numerical results of the experiment are as follows. Compared with the scheme that only adopts the no-signal priority strategy, the travel time for the comprehensive scheme in this work decreases by 16.60%, and the number of tram stops at junctions decreases by 53.66%. Compared with the scheme that adopts the active signal priority strategy during all travel periods, the negative utility of the signal priority for the comprehensive scheme proposed in this work is reduced by 39.45%. Overall, the comprehensive optimization scheme proposed in this work can minimize the travel time of the tram and the negative utility of signal priority for social vehicles.

This study provides an analysis and discussion of this model. However, the computation of the negative utility of signal priority for other social vehicles is simplified in this study. To be better implemented in actual tram lines, it is better to further explore the more detailed and accurate methods of quantifying the negative unit utility of different signal priority strategies in the model.

## Data Availability

The data that support the findings of this study are available from the corresponding author upon reasonable request.
